# The Colombo Twin and Singleton Study (COTASS): Piloting the Feasibility of Collecting Nutritional Data and Extension of the Sample to Include Children of Twins

**DOI:** 10.1007/s10519-023-10171-w

**Published:** 2024-01-07

**Authors:** Lasith Dissanayake, Binoli Herath, Janani Opatha, Sameeha Jabir, Rajindra Siriwardana, Kavish Sirisena, Malmi Wickramasinghe, Manouri Wimalasekera, Ruvini Liyanage, G. N. Duminda Guruge, Kaushalya Jayaweera, Ranil Jayawardena, Helena M. S. Zavos, Athula Sumathipala, Frühling Rijsdijk

**Affiliations:** 1 Institute for Research and Development in Health and Social Care, No. 393/3, Lily Avenue, Off Robert Gunawardena Mawatha, Battaramulla, Colombo, Sri Lanka; 2 Health and Wellness Unit, Faculty of Medicine, University of Colombo, Colombo, Sri Lanka; 3 Laboratory of Nutritional Biochemistry, National Institute of Fundamental Studies, Kandy, Sri Lanka; 4 Department of Health Promotion, Faculty of Applied Sciences, Rajarata University, Mihintale, Sri Lanka; 5 Department of Physiology, Faculty of Medicine, University of Colombo, Colombo, Sri Lanka; 6 Department of Psychology, Institute of Psychiatry, Psychology & Neuroscience, King’s College London, London, UK; 7 Faculty of Medicine & Health Sciences, Research Institute for Primary Care & Health Sciences, Keele University, Keele, UK; 8 Psychology Department, Faculty of Social Sciences, Anton de Kom University, Paramaribo, Suriname

**Keywords:** Nutrition intake, Psychological health, Intergenerational transmission, Diet, Twins, Sri Lanka

## Abstract

Nutrition and diet are key modifiable risk factors for the rising burden of non-communicable diseases like cardio-vascular diseases and diabetes in low- and middle- income countries (LMICs). The nutritional transition in dietary behaviours in LMICs has most likely contributed to this problem. Although traditionally assumed to be environmental, dietary choices are also genetically influenced. Twin study designs can be used to investigate the relative influence of genes and environment on nutrition intake, eating behaviours and associated psychological health. The overall aim of this project is to: provide proof-of-concept for the feasibility of using dietary (biomarker) data within the Children-of-Twin design in nutrition studies, develop laboratory skills and statistical genetic skills and establish a Sri Lankan-specific food composition database. Currently, a pilot study is being conducted with 304 individuals (38 Monozygotic twin pairs, 38 Dizygotic twin pairs and their male or female adult offspring). Questionnaire data on nutritional intake, eating behaviours, psychological well-being, physical health, and bio-specimens are being collected. A Sri Lankan-specific food composition database was developed, training sessions on macro and micro element analysis in biological samples and statistical genetics skills development were conducted and Community Engagement and Involvement programs were carried out in two districts of Sri Lanka.

## Introduction

The global burden of non-communicable diseases (NCDs) has increased and changes in diet are recognized as one of the leading contributors (Singh et al. 2020). Dietary patterns are influenced by a variety of social determinants including health literacy, socio-economic status, food preference, cultural diversity and food policies. Thus, the world has seen a remarkable shift in dietary behaviours that relate to the ‘nutrition transition’, where staple foods are becoming more refined and processed (Popkin 2015). Fat and meat intake is increasing, processed dairy products and other processed foods are consumed more often, and a larger number of meals are eaten outside the home (WHO 2002; Popkin 2015). Assessment of dietary intake among different populations is essential to monitor the ongoing nutritional transition and for the development of appropriate interventions (Jayawardena 2016).

NCDs are associated with unhealthy dietary patterns like excessive intake of sodium and processed foods (added sugars and unhealthy fats), and with a low intake of fruit and vegetables, whole grains, fibre, legumes, fish, and nuts (Casas et al. 2018). Historically, the Sri Lankan diet consisted of healthy foods involving vegetables, fruits, whole grains, legumes, and domestic tuber roots (Jayatissa et al. 2014). There has been a significant change in food consumption patterns in Sri Lanka which could be attributed to globalization (Weerasekara et al. 2018). Diet and nutrition are susceptible to influences within families (Birch et al. 2007), as eating is a social experience where parents transmit values to children to help them develop healthy eating habits. This influence can promote healthy as well as unhealthy dietary habits that predispose to risk of cardio-metabolic diseases and obesity. Besides this transmission of environmental risk/protection, dietary choice and nutrition are genetically influenced and genetically correlated with metabolic risk (Reed 1997; Teucher et al. 2007; Hasselbalch et al. 2008).

The ‘children of twins (CoT)’ design is applied in this study. The data collected from parents and children can be assessed through this design to examine the genetic and environmental influences on intergenerational transmission of human traits. The overall aim of this project is to use this CoT design to investigate the relationship between parental measures and child outcomes, and thereby assess the relative impact of transmitted genetic and environmental effects on nutrition and diet. The project also aims to construct a foundation for a food composition database (FCDB) which provides comprehensive nutritional information on Sri Lankan dishes. In addition, there are several training streams such as developing skills in macro and micro element analysis in biological samples, needed to validate the nutrition and dietary data assessed by questionnaires; statistical genetics skills development program to build capacity for twin data analysis; and training sessions on Community Engagement and Involvement (CEI), to train and establish a critical mass of people for future nutrition-related research in Sri Lanka.

## Methods and Analysis

### Pilot Study

#### Study Design

Complex traits such as dietary choice and nutrition are influenced by both genes and environment (Reed 1997; Teucher et al. 2007; Hasselbalch et al. 2008). However, there is a gap in knowledge on the intergenerational transmission of dietary choices and whether associations are due to direct environmental impact or transmitted genetic factors. Similarly, nutritional epidemiology needs an approach to control for confounding effects of environmental factors that are shared by family members (e.g., socio-economic status) (Ioannidis 2018).

To address this, quantitative genetic methods can be adopted to compare the similarity among family members and estimate the influence of genes and environment on their traits and behaviours. In addition, studying samples of twin pairs with children can provide valuable insight into the nature of intergenerational associations (McAdams et al. 2018). Therefore, a novel method in nutrition research is used in the present study, which is an extension of the classical twin model to include the children of the twins (CoT design).

#### Study Population and Setting

Participants of the COTASS-2 follow-up study (Jayaweera et al. 2018), who are currently living in the Colombo district of Sri Lanka, are invited for a Cross-Sectional study. Since this is a pilot study using an existing cohort, female twins (identical or non-identical) with adult offspring (age 18-years or above) from all ethnicities who speak Sinhala or English languages are purposively invited.

#### Exclusion Criteria

Twins are excluded if either one of them does not have an offspring aged 18-years or above, or if one of the twins or their offspring lives out of the Colombo district. Potential participants with cognitive impairments are also excluded.

#### Sample Size, Sampling Method, and Recruitment Process

The sample size was calculated considering the data collection period and available resources. Three hundred and four individuals (38 Monozygotic twin pairs, 38 Dizygotic twin pairs and their offspring) will be recruited for this study.

Potential participants are being contacted via phone by a research assistant (RA), and an information leaflet in English or Sinhala language is being given to the individuals who are interested in participating in the study. Informed written consent was obtained from the eligible twins and their offspring who participated in the study.

#### Questionnaire Data and Biospecimen Sample Collection, Transportation and Storage

Considering the COVID-19 safety concerns, the participants are given the option of completing the questionnaire by themselves and sending it via post or an RA to visit their houses. Data on diet, eating behaviours, mental health, general health, substance use and chronic diseases are being collected from all participants (see Table 1 for details). These questionnaires have been translated and culturally adapted for use in Sri Lanka. The food frequency questionnaire (Jayawardena et al. 2012, 2016), has been validated for use in Sri Lanka.

**Table 1:** Data collected for the study

Type of data	Questionnaire/biospecimen data	Description of data collected
Questionnaire data	Sociodemographic Questionnaire (Developed by the IRD)	Basic Socio-demographic details
	Eating Behaviour Questionnaire (Herle et al. 2019)	Eating Behaviours
	Beck Depression Inventory-II (Beck et al. 1961)	Presence and severity of depressive symptoms
	Generalized Anxiety Disorder (GAD-7) (Spitzer et al. 2006)	Presence and severity of anxiety symptoms
	Substance use (Nicotine and alcohol consumption) (Developed by the IRD)	Tobacco and Alcohol use screening tool
	Checklist of Chronic Disease (Developed by the IRD)	Presence of chronic diseases
	General Health (Short Form 36 Health Survey Questionnaire, SF-36) (McHorney et al. 1993)	Self-assessed general health
	Food Frequency Questionnaire (Jayawardena et al. 2012, 2016)	Frequency of the consumption of food types
	24-h Dietary Recall (Jayawardena 2016)	Details of diet during the past 24 h
Biospecimen data	Serum carotenoids	Correlate with fruit and vegetable intake Serum carotenoid content refers to the quantitative assessment of carotenoids present in the diet through the analysis of compounds in serum
	Serum macro mineral and ultra-trace mineral contents Macro minerals—Na, K, Mg, P, Cl, Ca Micro minerals—Fe, Cu, and Li	Correlate with all food intakes Minerals refer to the assessment of different macro and micro mineral contents in the diet. Food groups contain different types of minerals in different quantities
	Blood urea nitrogen (BUN)	Correlate with meat, fish, dairy and legume food intake Blood protein content refers to the quantitative assessment of protein in the diet
	Urinary total phenolic content	Correlate with fruit and vegetable intake Urinary total phenol content refers to the quantitative assessment of phenolic compounds present in the diet through the analysis of compounds excreted in the urine

In addition, 24-h dietary recall data (Jayawardena 2016) is being collected from participants before the biospecimen (blood and urine) collection. This allows to compare the 24-h dietary recall data and food frequency questionnaire data with serum and urinary markers for validation of subjective measures of dietary intake.

Blood and urine samples are being collected once on a convenient date for the participants by experienced phlebotomists working at an accredited private hospital. All current COVID-19 safety measures are being followed during the sample collection process. A total of 10 ml of blood and 20 ml of morning urine is collected after 8-h of fasting. Biospecimen is currently being analysed for the assessment of nutritional biomarkers.

Questionnaire data collected and biospecimen data analysed during the pilot study are indicated in Table 1.

## Data Analysis

### Model-Fitting Analysis

The specific analysis to generate the biospecimen variables are described in the next sections. These variables and the questionnaire data will be entered into SPSS statistical software (IBM SPSS Statistics 23), followed by a data cleaning process by trained data entry operators. Data will be analysed using SPSS and OpenMx within the R statistical software (Neale 2016). The statistical method used to estimate the effects of latent genetic and environmental factors on variances and covariances of traits; Structural Equation Model fitting (SEM), will be conducted using OpenMx. The models will vary from simple univariate twin models and bivariate twin models to more complex models including the novel Children-of-Twins model.

### Biospecimen Analysis

#### Analysis of Serum Carotenoid Content

Analysing serum carotenoid content and urinary total phenolic content as biomarkers is of great importance in gaining insight into individuals' fruit and vegetable intake. These compounds function as natural antioxidants within the human body, playing a crucial role in preventing various non-communicable diseases that many Sri Lankans suffer from due to poor dietary habits (Mennen et al. 2006; Medina-Remón et al. 2011). Serum carotenoid content will be analysed by the High-performance liquid chromatography (HPLC)-UV method with the universal C18 reversed-phase column (Gueguen et al. 2002; Tremblay et al. 2018).

#### Analysis of Serum Macro Mineral and Ultra-Trace Mineral Content

The mineral composition reflects the integration of these diverse varieties into one's diet. Both macro and micro minerals hold significant importance in supporting a wide range of physiological functions within the human body. Their pivotal roles include promoting proper growth, facilitating development, and ensuring overall well-being (Livingstone and Black 2003).

Macro minerals; sodium (Na), potassium (K), magnesium (Mg), phosphorus (P), chlorine (Cl), calcium (Ca), and micro minerals; iron (Fe), copper (Cu), and lithium (Li) will be determined using the following methods in Olympus AU 400 auto analyser and Electrolyte Analyser/ AVL machine by an appointed medical laboratory service provider (Duffy 1982; Khalil et al. 1986; Burnett et al. 2000; Löwe et al. 2005) (Table 2).

**Table 2:** Biospecimen analysis methods for determining serum mineral content

Cl, Na, K, Li	Ion-selective electrode (ISE) method
Ca	Colorimetric endpoint assay
Mg	Magnesium assay kit
P	Colorimetric blue of molybdate method
Fe	FerroZine method
Cu	3,5-Di Br Paesa method
Blood urea nitrogen (BUN)	Colorimetric kinetic method
Urinary total phenolic content (TPC)	Fast Blue BB method

### Determination of Blood Urea Nitrogen (BUN)

Nitrogen serves as an indicator of protein consumption. The intake of proteins holds paramount importance for the human body, as they serve numerous essential functions that greatly contribute to maintaining overall health and well-being (Peng et al. 2023). These roles encompass a wide array of physiological processes that are vital for optimal functioning. Protein-rich sources primarily include meat, fish, dairy products, and legumes. Determination of BUN will be carried out according to the colorimetric kinetic method in the Olympus AU 400 auto analyser.

### Determination of Urinary Total Phenolic Content

Polyphenols are non-nutritive plant secondary metabolites commonly found in the human diet. Both experimental and epidemiological data have suggested the role of polyphenols in the prevention of chronic diseases, particularly cardiovascular diseases, type-2 diabetes and certain cancers (Vauzour et al. 2010; Huang et al. 2018). Urinary Total Phenolic Content (TPC) will be assessed by the Fast Blue BB method (Hinojosa-Nogueira et al. 2017).

### Development of a Sri Lankan Specific Food Composition Database

#### Selecting the List of Food Items to be Included in the FCDB

Four different approaches were used to select the list of food items. Foods that are commonly consumed by the Sri Lankan population were selected using the national level dietary survey from the development phase of the Food Frequency Questionnaire (FFQ) for Sri Lankan adults (Jayawardena et al. 2013). Some leading supermarkets and a few fast-food outlets were visited. Marketing personnel of these outlets were interviewed to get records of the popular food items available in the supermarkets or fast-food outlets. Data was also acquired from local literature.

#### Assigning Food Groups, Names and Codes

The list of food items was categorized into food groups. Food names and food codes were assigned according to the International Network of Food Data Systems (INFOODS) regulations (FAOUN 2022).

#### Compiling Food Composition Data (FCD)

FCD for raw food commodities were assembled from existing FCDBs; primarily the United States Department of Agriculture (USDA) Food Composition Database (USDA 2019), the United Kingdom (UK) Food Composition Database (McCance and Widdowson 2015), and Sri Lankan Food Composition Tables (FCTs) (Jayatissa et al. 2021). We also used original research articles to acquire FCD. Food composition was calculated using the recipe calculation method.

FCD for cooked food was assessed through two different methods. (1) FCD was calculated based on cooking conversion factors in food commodities for which cooking conversion factors are published through sufficient research (Adikari and Thamilini 2018). (2) Food commodities were cooked/prepared using standard recipes for which conversion factors were not published. The recipe collection was done using recipe books that contained Sri Lankan household recipes.

Conversion factors; which are the ratio, and weights of cooked edible portions to raw edible portions, were calculated based on the measurements. Recipe calculation was performed to determine FCD. Information about edible portions, weight changes resulting from fat and water uptake or loss (conversion factors), and retention factors were required for recipe calculation (Bognár and Piekarski 2000).

In this method, all the ingredients were recorded with their weight in grams. The weight of each ingredient was summed up and the result was multiplied by their conversion factor. This was multiplied by the nutrient retention factor for each nutrient (USDA 2019). Yield factors for fat change were applied at the recipe level (Reinivuo and Laitinen 2007).

FCD for branded foods were recorded from food labels. Food companies were contacted directly if the food composition data were not presented in labels.

## Ethical Considerations and Dissemination

### Consent and Information Provision

Permission to conduct a community-based research study during the COVID-19 epidemic was obtained from the Ministry of Health, Sri Lanka. After the initial communication with the potential participants, an information leaflet and consent form of preferred language are either hand delivered or posted. Adequate time is given to read and understand the information and if necessary, to discuss it with family members or relatives. They are also informed regarding voluntary participation and the right to withdraw from the study or any component of the study. Participants can withdraw from the study before, during and up to one week after the interview date without having to explain why and without any penalty.

To avoid undue inducement, we do not offer large incentives or compensations, but considering the time/travelling cost, and participants who prefer visiting biospecimen collection centres are offered a reasonable compensation.

### Data Storage, Sharing, Access, Confidentiality and Anonymity

Participants of the COTASS longitudinal study were assigned a unique identification number, and this will be used for identification purposes in this pilot study. Any personal information of the participants is managed on a strictly confidential basis and not be divulged to third parties. All confidentiality arrangements adhere to relevant regulations and guidelines in the UK Data Protection Act (UK Legislation 1998).

#### Questionnaire Data

Personal details of participants and documents that contain non-anonymised data is stored in a confidential, password-protected database, only accessible to the research team. Non-anonymised data will be destroyed and the anonymised data will be kept for possible future use for a period of up to 10 years.

#### Biospecimen Data

Only the unique identification number with contact details of the participants are being disclosed to the field sample collection team as well as the data analysis teams. Following the analysis, all remaining blood and urine samples will be discarded after mixing with 80% ethanol. The standard operating procedures for the safe disposal of infectious laboratory waste will be followed in the laboratories.

## Discussion

### Challenges Encountered During the Study

We encountered several challenges as the project was undertaken during the COVID-19 pandemic. The data collection process was delayed due to restrictions on movement/travel and physical distancing. Questionnaires were posted to participants who did not prefer house visits. These participants were guided via phone about filling out the questionnaire. Compared to previous COTASS studies, the field data collection team experienced a lot of difficulties in recruiting potential participants. Many of them were initially reluctant to take part due to fear of contracting the COVID-19 virus. Following the COVID-19 pandemic, Sri Lanka faced adversity due to a severe economic crisis (World Bank 2022). This resulted in high inflation, a shortage of medical and essential supplies, and increased prices of basic commodities. We believe these economic hardships and related consequences have further discouraged potential participants from taking part in the study. Although, global academic research activities have been hampered significantly owing to these reasons (Haleem et al. 2020; Harper et al. 2020), we have been able to continue our work progressively coping successfully, and we believe that reporting the unique perspectives and experiences of this research team would benefit fellow researchers to plan their research ahead to meet the future demands proactively.

Recruitment of participants, which includes twin mothers with adult offspring (aged 18 years or above), is a challenging task. Most twin mothers in the COTASS sample have children below 18 years and therefore had to be excluded. Among the eligible study units (one study unit includes four individuals: twin mothers, and one adult offspring of each twin mother) some participants were unavailable for the study due to migration, living apart from or being employed out of the Colombo district.

## Research Collaborations

The Institute for Research and Development in Health and Social Care (IRD) as an academic research institution, has pioneered in conducting health and social care research, through a team of long-term collaborators; local, regional and countries from the global north who have expertise in conducting high-quality research. In addition to the established long-term collaboration between researchers at Kings College London and Keele University UK, IRD plans to develop and extend new collaborative links with (1) The National Institute of Fundamental Studies (NIFS), a premier multidisciplinary research institute that facilitates fundamental and advanced scientific research in Sri Lanka and (2) The Department of Health Promotion, Rajarata University of Sri Lanka which has long-standing experience in developing and utilizing a ‘community-based health promotion approach.

### Sri Lankan-Specific Food Composition Database (FCDB)

FCDBs are developed to support health professionals and individuals in clinical and public health-level disease management. FCDBs are fundamental for nutrition science and are used extensively in the public health domain, providing detailed information on the nutritional composition of foods that are important for the country’s nutrition profile. The first FCT in Sri Lanka was published in 1979 by the Department of Nutrition, Medical Research Institute (MRI) (Perera et al. 1979; Thamilini et al. 2015). Later it was updated and published as a more comprehensive book in 2021 (Jayatissa et al. 2021), which contains FCD for raw food commodities, but it lacks nutritional information on cooked food, mixed dishes and ready-to-eat foods that can be purchased off the shelf. Up to date, FCDs are available in several countries including the United States (Ahuja et al. 2013), countries in South East Asia (Puwastien 2000) and India (Longvah et al. 2017).

A food exchange list is a system that determines the daily food plan based on units or exchanges of various food types, specific to different cultures and practices followed by different groups of people (Khan et al. 2017). Therefore, a multi-ethnic country like Sri Lanka would have a wide variation. Hence developing an up-to-date electronic FCDB would allow us to determine the daily food plan according to the present context, and also support future nutritional research conducted in Sri Lanka and the South Asian region.

### Dietary Biomarkers for Dietary Validation

Empirical research has reported the significant influence of genetics on various food preferences and dietary habits (Teucher et al. 2007). However, a substantial research gap exists in understanding the genetic impact on food choices within the unique context of Sri Lanka. This discrepancy arises from the distinctiveness of Sri Lankan culinary traditions compared to those of other nations. As a result, this study holds immense importance in unravelling the genetic effects on diverse food categories and their corresponding dietary biomarkers.

Dietary biomarkers serve as measurable indicators within the body, offering valuable insights into an individual's dietary consumption and dietary patterns. This insight is achieved through the analysis of biospecimens. Biospecimen analysis involves laboratory procedures that reveal information for dietary validation. The process of dietary validation is done through statistical analysis, considering both dietary assessments/questionnaires and biospecimen data (Kuhnle 2012).

### Capacity Development: Statistical Genetics Skills Development

Training the next generation of twin researchers in Sri Lanka in the Twin method and the Children-of-Twins design is essential for capacity building. Developing models for twin data analysis requires specialized training. Currently, there is only one international course on twin data analysis: The Boulder Statistical Genetics Workshop, University of Colorado. As most twin studies are still conducted in high-income countries, this course is mostly attended by EU and US delegates. However, with the rise of twin studies conducted in LMIC, it is important to make this training available to different parts of the world. So, a Statistical Genetics course was conducted by the researchers from King’s College London to build capacity in twin data analysis and interpretation, among the Sri Lankan researchers using OpenMx in R statistical software. This training will also support the utilization of existing Colombo Twin and Singleton Study (COTASS) data resources.

### Community Engagement and Involvement (CEI)

Community Engagement and Involvement (CEI) in research refers to a process of active partnership between members of the community and researchers (NIHR 2023). During the process, a group of community members are involved and engaged in various stages of the research project (Hoddinott et al. 2018; NIHR 2019). This enables research being carried out ‘with’ or ‘by’ members of the public rather than ‘to’, ‘about’ or ‘for’ them”. The contribution of the CEI group improves the relevance, accountability and transparency of research.

Despite the emerging trend in CEI in research, utilization of this approach in health research is limited in South Asia. CEI, a relatively novel concept to Sri Lanka, is not yet well developed and incorporated in research. To bridge this gap in knowledge, a training component in CEI was integrated and applied in the pilot project.

#### Training a CEI Group in Nutrition Research

The IRD, in partnership with Keele University, UK, is in the process of establishing a strong and visible CEI pillar in Sri Lanka. This is done by building a cooperative network with multiple stakeholders, with the aim of sharing knowledge and practices of CEI activities with the wider public, broadening the opportunities and identifying challenges for CEI. This initiative will be further developed to establish a research collaboration with the Department of Health Promotion, Rajarata University of Sri Lanka.

This training focused on establishing a critical mass of people (3 different groups); (i) senior, mid-career and early career researchers on the project (ii) researchers/academics outside the project team (iii) a new project specific non-academic lay group at the Rajarata University of Sri Lanka, to support future nutritional research. Training provided on key aspects of CEI, covering values and principles of CEI, methodological aspects, practical and ethical considerations, and quality components. The training was based upon international frameworks for community engagement (e.g., UNICEF’s Minimum Quality Standards and Indicators for Community Engagement; UK Public Involvement Standards). The training was delivered and adapted appropriately according to the needs of the three groups, specifically ensuring that clear and plain language and resources are used for the public groups.

## Strengths and Limitations of the Study

This project is the first step taken to develop a unique Sri Lankan-specific food composition database as Sri Lanka lacked an inclusive food composition database adapted to the local context. This addresses the gap in knowledge on food composition of commonly consumed food items including both cooked and mixed dishes. The outcomes of this project are invaluable for future nutrition research as well as in fostering good nutrition.

For resource-poor LMIC settings, the development of social, educational, technological, and economic domains is crucial, in order to overcome the NCD burden and under-nourishment, which primarily depends on research evidence (Siriwardhana 2015). However, specific resources supporting the research activity such as opportunities for training and capacity building for researchers are limited. This project intends to address this concern by setting up three key training streams while the pilot study will provide a ‘proof of concept’ of using dietary (biomarker) data within the Children-of-Twins design in nutrition studies. The results will also help to create a high-quality research base for nutrition health within Sri Lanka.

Furthermore, the exchange of new scientific knowledge with the wider global scientific community is essential for improving quality, increasing quantity, and ensuring continuity. Research collaborations enable comparison, contrasting, critique, and dialogue between peers from similar settings and more “resource-rich” settings. Research partnerships built through this project, will also facilitate increased attention to LMIC research, generate funding, and promote future East–West collaborations.

However, our work clearly has some limitations. We acknowledge the fact that study findings from a twin cohort of the Colombo district in Sri Lanka might be different to that of other areas of the country, and also may not be generalizable to other LMICs, because of the varying impact of heritable and environmental factors, which are population-specific. However, as there is a good representation of ethnic minorities within this cohort, this methodology could potentially be adopted in similar settings. Another possible limitation is that the nutritional data is being collected during an economic crisis, which could have affected people’s nutritional choices.

## Conclusion

The present study was designed to lay the foundation for future nutrition-related research in Sri Lanka. This includes the transfer and development of knowledge to conduct nutrition-related biomarker analyses as well as statistical (genetics) analyses. Further, this study is the first to develop a Sri Lankan-specific food composition database as a reference for future nutrition research. In addition, valuable insights will be gained in terms of intergenerational transmission of nutrition-related risk/protective factors.

## Data Availability

Collected data is stored at the Institute for Research and Development, Sri Lanka. Individual Researchers / Organizations who are interested in using this data, can make an official request through info@ird.lk and obtain the data.

## References

[CR1] Adikari A, Thamilini J (2018). Cooking conversion factor of commonly consumed Sri Lankan food items. MOJ Food Process Technol.

[CR2] Ahuja JKC (2013). USDA food and nutrient databases provide the infrastructure for food and nutrition research, policy, and practice. J Nutr.

[CR3] Beck AT (1961). An inventory for measuring depression. Arch Gen Psychiatry.

[CR4] Birch L, Savage JS, Ventura A (2007). Influences on the development of children’s eating behaviours: from infancy to adolescence. Can J Dietetic Pract Res.

[CR5] Bognár A, Piekarski J (2000). Guidelines for recipe information and calculation of nutrient composition of prepared foods (Dishes). J Food Compos Anal.

[CR6] Burnett RW (2000). Use of ion-selective electrodes for blood-electrolyte analysis: recommendations for nomenclature, definitions and conventions. Clin Chem Lab Med.

[CR7] Casas R (2018). Nutrition and cardiovascular health. Int J Mol Sci.

[CR8] Duffy JR (1982). A fully automated method for determining total serum iron and unsaturated ironbinding capacity using a technicon AAII auto analyser system. Clin Biochem.

[CR9] FAOUN (2022) International network of food data systems: training. https://www.fao.org/infoods/infoods/training/en/. Accessed 17 April 2023

[CR10] Gueguen S (2002). An isocratic liquid chromatographic method with diode-array detection for the simultaneous determination of α-tocopherol, retinol, and five carotenoids in human serum. J Chromatogr Sci.

[CR11] Haleem A (2020). Areas of academic research with the impact of COVID-19. Am J Emerg Med.

[CR12] Harper L (2020). The impact of COVID-19 on research. J Pediatr Urol.

[CR13] Hasselbalch AL (2008). Studies of twins indicate that genetics influence dietary intake. J Nutr.

[CR14] Herle MP (2019). The association between emotional eating and depressive symptoms: a population-based twin study in Sri Lanka. Global Health, Epidemiol Genom.

[CR15] Hinojosa-Nogueira D (2017). New method to estimate total polyphenol excretion: comparison of fast blue BB versus Folin-Ciocalteu performance in urine. J Agric Food Chem.

[CR16] Hoddinott P (2018). How to incorporate patient and public perspectives into the design and conduct of research. F1000Research.

[CR17] Huang J (2018). Serum beta carotene and overall and cause-specific mortality: a prospective cohort study. Circ Res.

[CR18] Ioannidis JPA (2018). ‘The challenge of reforming nutritional epidemiologic research. J Am Med Assoc.

[CR19] Jayatissa R, Wickramasinghe W, Piyasena C (2014) Food consumption patterns in Sri Lanka, Food Consumption Pat-terns in Sri Lanka. Hector Kobbekaduwa Agrarian Research and Training Institute. https://harti.gov.lk/images/download/reasearch_report/new1/172.pdf

[CR20] Jayatissa R et al. (2021) Sri Lanka Food Composition Tables (SLFCT), 2nd edn. Colombo: Department of Nutrition, Medical Research Institute, Ministry of Health. https://www.mri.gov.lk/units/nutrition/survey-reports/

[CR21] Jayawardena R (2016). Comparison dietary assessment methods in Sri Lankan adults: use of 24-hour dietary recall and 7-day weighed intake. BMC Nutr.

[CR22] Jayawardena R (2012). Development of a food frequency questionnaire for Sri Lankan adults. Nutr J.

[CR23] Jayawardena R (2013). Food consumption of Sri Lankan adults: an appraisal of serving characteristics. Public Health Nutr.

[CR24] Jayawardena R (2016). Validity of a food frequency questionnaire to assess nutritional intake among Sri Lankan adults. Springerplus.

[CR25] Jayaweera K (2018). The Colombo twin and singleton follow-up study: a population based twin study of psychiatric disorders and metabolic syndrome in Sri Lanka. BMC Public Health.

[CR26] Khalil SAH, Moody GJ, Thomas JDR (1986). Ion-selective electrode determination of sodium and potassium in blood and urine. Anal Lett.

[CR27] Khan MN, Kalsoom S, Khan AA (2017). Food exchange list and dietary management of non-communicable diseases in cultural perspective. Pak J Med Sci.

[CR28] Kuhnle GGC (2012). Nutritional biomarkers for objective dietary assessment. J Sci Food Agric.

[CR29] UK Legislation (2023) ‘Data Protection Act 2018’. In: Data protection and data transfers law. Bloomsbury Professional. 10.5040/9781526524812.schedule-003

[CR30] Livingstone MBE, Black AE (2003). Markers of the validity of reported energy intake. J Nutr.

[CR31] Longvah T. et al. (2017) Indian food composition tables. National Institute of Nutrition. http://www.ifct2017.com/frame.php?page=home. Accessed 16 April 2023

[CR32] Löwe A, Balzer T, Hirt U (2005). Interference of gadolinium-containing contrast-enhancing agents with colorimetric calcium laboratory testing. Invest Radiol.

[CR33] McAdams TA (2018). Revisiting the children-of-twins design: improving existing models for the exploration of intergenerational associations. Behav Genet.

[CR34] McCance and Widdowson (2015) Composition of foods integrated dataset (CoFID)—GOV.UK. PHE publications gateway number: GW–2010. https://www.gov.uk/government/publications/composition-of-foods-integrated-dataset-cofid. Accessed 19 Aug 2023

[CR35] McHorney CA, Ware JE, Raczek AE (1993). The MOS 36-item short-form health survey (Sf-36): II—psychometric and clinical tests of validity in measuring physical and mental health constructs. Med Care.

[CR36] Medina-Remón A (2011). Total polyphenol excretion and blood pressure in subjects at high cardiovascular risk. Nutr Metab Cardiovasc Dis.

[CR37] Mennen LI (2006). Urinary flavonoids and phenolic acids as biomarkers of intake for polyphenol-rich foods. Br J Nutr.

[CR38] Neale MC (2016). OpenMx 2.0: extended structural equation and statistical modeling. Psychometrika.

[CR39] NIHR (2019) Ways that people can be involved in the research cycle. https://www.invo.org.uk/posttyperesource/where-and-how-to-involve-in-the-research-cycle/. Accessed 3 July 2022

[CR40] NIHR (2023) Engage and involve communities. https://www.nihr.ac.uk/researchers/apply-for-funding/how-to-apply-for-global-health-funding/community-engagement-and-involvement.htm. Accessed 17 April 2023

[CR41] Peng X (2023). Serum nutritional biomarkers and all-cause and cause-specific mortality in U.S. adults with metabolic syndrome: the results from national health and nutrition examination survey 2001–2006. Nutrients.

[CR42] Perera W, Jayasekera, Thaha S (1979) Tables of food composition for use in Sri Lanka. Colombo: World Health Foundation of Sri Lanka. https://agris.fao.org/agris-search/search.do?recordID=XF2015028527

[CR43] Popkin BM (2015). Nutrition transition and the global diabetes epidemic. Curr Diab Rep.

[CR44] Puwastien P (2000). Food composition programme of ASEANFOODS 1995–1999. J Food Compos Anal.

[CR45] Reed DR (1997). Heritable variation in food preferences and their contribution to obesity. Behav Genet.

[CR46] Reinivuo H, Laitinen K (2007) Proposal for the harmonisation of recipe calculation procedures—WP2.2 composite foods. https://www.intake.org/resource/proposal-harmonisation-recipe-calculation-procedures

[CR47] Singh JE (2020). Mapping the global evidence on nutrition transition: a scoping review protocol. BMJ Open.

[CR48] Siriwardhana C (2015). Promotion and reporting of research from resource-limited settings. Infect Dis.

[CR49] Spitzer RL (2006). A brief measure for assessing generalized anxiety disorder: the GAD-7. Arch Intern Med.

[CR50] Teucher B (2007). Dietary patterns and heritability of food choice in a UK female twin cohort. Twin Res Hum Genet.

[CR51] Thamilini J (2015). Food composition data in Sri Lanka: past, present and future.

[CR52] Tremblay BL (2018). ‘Genetic and common environmental contributions to familial resemblances in plasma carotenoid concentrations in healthy families. Nutrients.

[CR53] USDA (2019) Food data central. https://fdc.nal.usda.gov/. Accessed 19 Aug 2023

[CR54] Vauzour D (2010). Polyphenols and human health: prevention of disease and mechanisms of action. Nutrients.

[CR55] Weerasekara PC (2018). Nutrition transition and traditional food cultural changes in Sri Lanka during colonization and post-colonization. Foods.

[CR56] WHO (2002) Globalization, diets and noncommunicable diseases. http://www.who.int/iris/handle/10665/42609

[CR57] World Bank (2022) The World Bank in Sri Lanka. https://www.worldbank.org/en/country/srilanka/overview

